# Deep Learning Algorithms for the Detection of Suspicious Pigmented Skin Lesions in Primary Care Settings: A Systematic Review and Meta-Analysis

**DOI:** 10.7759/cureus.65122

**Published:** 2024-07-22

**Authors:** Ahmed R Abdalla, Ahmed W Hageen, Haneen H Saleh, Omar Al-Azzawi, Mahmoud Ghalab, Amani Harraz, Bola S Eldoqsh, Fatma E Elawady, Ayman H Alhammadi, Hesham Hassan Elmorsy, Majd Jano, Mohamed Elmasry, Eshak I Bahbah, Ahmed Elgebaly

**Affiliations:** 1 Vascular Surgery, Faculty of Medicine, Mansoura University, Mansoura, EGY; 2 Artificial Intelligence Research Group, MedDots Academy, Cairo, EGY; 3 Faculty of Medicine, Tanta University, Tanta, EGY; 4 Faculty of Medicine, University of Jordan, Amman, JOR; 5 Faculty of Pharmacy, İstinye University, İstanbul, TUR; 6 Radiology Department, Kafrelsheikh University, Kafr El Sheikh, EGY; 7 Faculty of Medicine, Alexandria University, Alexandria, EGY; 8 Faculty of Medicine, Minia University, Minia, EGY; 9 Department of Ophthalmology, Port Said Specialized Hospital of Ophthalmology, Port Said, EGY; 10 Department of Radiology, Faculty of Medicine, Alexandria University, Alexandria, EGY; 11 Faculty of Pharmacy, Helwan University, Helwan, EGY; 12 Research Department, Syrian Society for Physicians and Pharmacists, Frankfurt, DEU; 13 Faculty of Medicine, Al-Azhar University, Damietta, EGY; 14 Smart Health Centre, University of East London, London, GBR

**Keywords:** screening, detection, melanoma, deep learning, artificial intelligence

## Abstract

Early detection of suspicious pigmented skin lesions is crucial for improving the outcomes and survival rates of skin cancers. However, the accuracy of clinical diagnosis by primary care physicians (PCPs) is suboptimal, leading to unnecessary referrals and biopsies. In recent years, deep learning (DL) algorithms have shown promising results in the automated detection and classification of skin lesions. This systematic review and meta-analysis aimed to evaluate the diagnostic performance of DL algorithms for the detection of suspicious pigmented skin lesions in primary care settings. A comprehensive literature search was conducted using electronic databases, including PubMed, Scopus, IEEE Xplore, Cochrane Central Register of Controlled Trials (CENTRAL), and Web of Science. Data from eligible studies were extracted, including study characteristics, sample size, algorithm type, sensitivity, specificity, diagnostic odds ratio (DOR), positive likelihood ratio (PLR), negative likelihood ratio (NLR), and receiver operating characteristic curve analysis. Three studies were included. The results showed that DL algorithms had a high sensitivity (90%, 95% CI: 90-91%) and specificity (85%, 95% CI: 84-86%) for detecting suspicious pigmented skin lesions in primary care settings. Significant heterogeneity was observed in both sensitivity (p = 0.0062, I^2^ = 80.3%) and specificity (p < 0.001, I^2^ = 98.8%). The analysis of DOR and PLR further demonstrated the strong diagnostic performance of DL algorithms. The DOR was 26.39, indicating a strong overall diagnostic performance of DL algorithms. The PLR was 4.30, highlighting the ability of these algorithms to influence diagnostic outcomes positively. The NLR was 0.16, indicating that a negative test result decreased the odds of misdiagnosis. The area under the curve of DL algorithms was 0.95, indicating excellent discriminative ability in distinguishing between benign and malignant pigmented skin lesions. DL algorithms have the potential to significantly improve the detection of suspicious pigmented skin lesions in primary care settings. Our analysis showed that DL exhibited promising performance in the early detection of suspicious pigmented skin lesions. However, further studies are needed.

## Introduction and background

Skin cancer is one of the most common cancers worldwide, with an estimated 1.5 million new cases diagnosed each year [1]. Among the different types of skin cancer, melanoma is the most aggressive tumor, accounting for approximately 75% of skin cancer-related deaths [2]. The burden of skin cancers is expected to increase further due to population growth, aging, and increased exposure to ultraviolet radiation [1]. Early detection and diagnosis of skin cancer, particularly melanoma, is crucial for improving patient outcomes and survival rates. The five-year survival rate for melanoma patients with localized disease is over 99%, but it drops dramatically to 74% for regional disease and 35% for distant metastasis [3]. A significant proportion of skin cancers are diagnosed at advanced stages, leading to worse prognoses and increased healthcare costs [4].

Primary care physicians (PCPs) are often the first point of contact for patients with suspicious skin lesions. In primary care settings, visual inspection of patients to identify suspicious pigmented lesions is a well-established dermatological practice. The ABCDE criteria, which include asymmetry, border unevenness, color distribution, diameter, and evolution, have been traditionally used for early-stage melanoma screening by PCPs [5]. However, recent research suggests that highly accurate and skilled clinical detection of melanoma relies more on unconscious visual pattern recognition and "ugly duckling" comparisons than on the simplified ABCDE criteria. However, the accuracy of clinical diagnosis by PCPs is suboptimal, with studies reporting sensitivities ranging from 55% to 73% [6,7]. This can result in unnecessary referrals and biopsies, causing anxiety and stress for patients and increasing the burden on healthcare systems [8]. Therefore, there is a pressing need for more accurate and efficient diagnostic tools to aid PCPs in the early detection and diagnosis of skin cancers.

In recent years, artificial intelligence (AI) has shown great promise in improving the diagnosis and management of various medical conditions, including skin cancers [9]. Deep learning (DL) is a subset of machine learning (ML) that uses artificial neural networks to model and solve complex problems. These algorithms can automatically learn and extract relevant features from large datasets, enabling them to identify patterns and make predictions with high accuracy [10]. In the context of skin cancer, DL algorithms have been applied to the analysis of clinical images to improve the diagnostic accuracy of skin lesions compared to naked-eye examination alone by providing automated and objective analysis of dermoscopic images. Several studies have demonstrated the efficacy of DL algorithms in detecting and classifying skin lesions, including melanoma, with high sensitivity and specificity [11]. Nonetheless, these studies evaluated DL algorithms for dermoscopic images, which require specialized training and expertise that are not readily available in primary care settings [11].

In recent years, advances in smartphone technologies have increased access to high-quality personal cameras and robust mobile computing systems, which have been applied to dermatology using computer-aided diagnosis (CAD) systems [12]. Recent studies have evaluated the usefulness of employing DL algorithms in next-generation CAD systems for the evaluation of suspicious pigmented lesions in primary care settings. These studies have shown that DL-based models can achieve comparable or even superior diagnostic accuracy to board-certified dermatologists in visual inspection [13,14]. However, the diagnostic performance of DL algorithms for the detection of suspicious pigmented skin lesions in primary care settings has not been systematically evaluated.

Therefore, this systematic review and meta-analysis aimed to evaluate the diagnostic accuracy of DL algorithms for the detection of suspicious pigmented skin lesions in primary care settings.

## Review

Methods

The present systematic review and meta-analysis were prepared in concordance with the 2020 Preferred Reporting Items for Systematic Reviews and Meta-Analyses (PRISMA) guidelines [15].

Search Strategy

A systematic literature search was conducted across multiple electronic databases, including MEDLINE via PubMed, Scopus, IEEE Xplore, Cochrane Central Register of Controlled Trials (CENTRAL), and Web of Science databases. The search strategy for PubMed and IEEE Xplore were as follows: (((((Learning, Deep OR Hierarchical Learning) AND (Intelligence, Artificial OR Computational Intelligence OR Machine Intelligence OR Computer Reasoning OR Computer Vision Systems OR Computer Vision System OR Knowledge Acquisition) AND (machine learning OR Transfer Learning) AND (skin lesions OR skin cancer OR Skin Neoplasm) AND (melanoma OR Melanomas OR Malignant Melanoma))))). For the remaining databases, we employed the following search strategy: (Computer Vision Systems OR Deep learning) AND (skin cancer) AND (melanoma OR Malignant Melanoma).

Eligibility Criteria and Screening

We included all studies that utilized DL algorithms for the detection of suspicious pigmented skin lesions in primary care settings. We limited our inclusion to studies that reported sufficient data to calculate the diagnostic accuracy of DL algorithms, including true positives, false positives, true negatives, and false negatives. Studies that were non-English, case reports, case series, review articles, editorial comments, letters, and studies that focused on the use of DL algorithms outside of the primary care setting or for non-pigmented lesions were excluded.

A two-step screening process was conducted to identify studies that met the predefined inclusion criteria. First, two reviewers independently screened the titles and abstracts of all retrieved studies to identify potentially eligible studies. Then, the full texts of the potentially eligible studies were obtained and assessed independently by the same two reviewers to determine their eligibility for inclusion. Disagreements between the reviewers were resolved by discussion to reach a final decision. If a consensus could not be reached, a third reviewer was consulted to make a final decision.

Data Extraction and Quality Assessment

Data extraction was performed systematically to extract relevant information from the included studies. The data extraction process was conducted by three reviewers who independently extracted data from each included study and recorded it in a predesigned Excel sheet (Microsoft Corporation, Redmond, WA). The extracted data included authors, year of publication, country, study design, demographic characteristics of the population, type of the lesion investigated, DL algorithms characteristics, and their sensitivity, specificity, positive predictive value, negative predictive value, and area under the receiver operating characteristic (ROC) curve.

The risk of bias assessment of the included studies was conducted using the Quality Assessment of Diagnostic Accuracy Studies 2 (QUADAS-2) score to evaluate the methodological quality and applicability of primary diagnostic accuracy in the included studies [16]. The risk of bias was assessed using four domains: patient selection, index test, reference standard, flow, and timing. Studies were rated as "low risk of bias," "high risk of bias," or "unclear risk of bias."

Data Analysis

The statistical analysis was conducted using the meta package in R software version 4.1.2 (R Foundation for Statistical Computing, Vienna, Austria). We performed a bivariate random-effects meta-analysis model to synthesize sensitivity, specificity, positive likelihood ratio (PLR), negative likelihood ratio (NLR), and diagnostic odds ratios (DOR) from the included studies. The diagnostic performance across studies was summarized using the summary receiver operating characteristic (SROC) curve, along with the calculation of the area under the curve (AUC). Heterogeneity among the included studies was assessed using the I² statistic, where values of 25%, 50%, and 75% represent low, moderate, and high heterogeneity, respectively. Cochran's Q test was also used to evaluate the statistical significance of observed heterogeneities. A p-value of less than 10% represented significant heterogeneity.

Results

Literature Search Results

**Figure 1:**
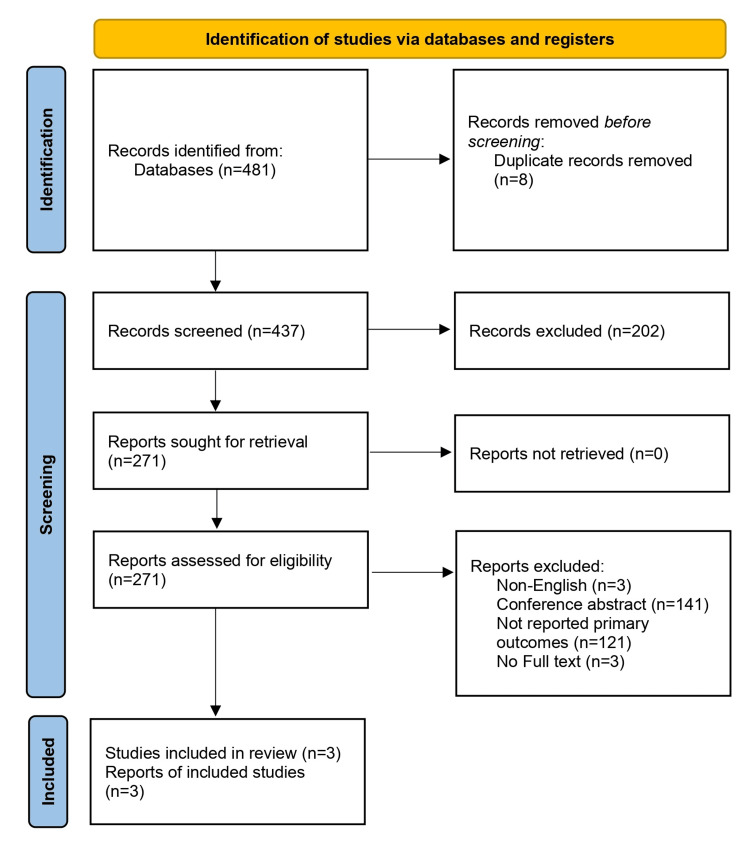
PRISMA 2020 flow diagram. PRISMA: Preferred Reporting Items for Systematic Reviews and Meta-Analyses.

A total of 473 unique records were identified through the literature search. A total of 202 records were excluded during the initial screening phase, and 271 full-text articles were assessed for eligibility. Of them, three studies were included in the present systematic review and meta-analysis (Figure 1) [12,14,17].

Characteristics of the Included Studies

Three studies employed DL techniques to detect pigmented skin lesions in primary settings. Sangers et al. conducted a prospective diagnostic accuracy study using a convolutional neural network (CNN, version RD-174). The study involved 372 participants with a median age of 71 years (range = 58-78), and approximately half were males (49.2%). Out of the 785 skin lesions assessed, 275 were identified as premalignant or malignant and 510 as benign. The main finding was that the AI-powered, commercially available application exhibited high sensitivity and specificity in detecting suspicious lesions [17]. Birkenfeld et al. also carried out a prospective diagnostic accuracy study but used logistic regression combined with principal component analysis (PCA). The sample included 133 individuals, predominantly male (54.13%), with ages ranging from 16 to 76 years. The study analyzed a total of 1759 lesions, distributed between a training set of 1187 lesions and a test set of 572 lesions. The results highlighted that the DL-powered CAD system could efficiently differentiate between suspicious and non-suspicious lesions [12]. Soenksen et al. utilized a deep convolutional neural network (DCNN) in a retrospective diagnostic accuracy study involving a large dataset of 33,980 images, which included 4,063 suspicious pigmented lesions (SPLs). Although specific demographic details were not provided, the study concluded that the DL-powered tool enabled accurate detection of SPLs within a primary care setting [14], as shown in Table 1.

**Table 1:** Summary characteristics of the included studies. DCNN: deep convolutional neural network; PCA: principal component analysis; CNN: convolutional neural network; DL: deep learning; PPV: positive predictive value; NPV: negative predictive value; NR: not reported.

Authors, year	Country	Study design	DL algorithm	Minimum specifications	Sensitivity, % (95% CI)	Specificity, % (95% CI)	PPV, % (95% CI)	NPV, % (95% CI)	AUC, % (95% CI)	Accuracy, % (95% CI)
Sangers et al. (2022) [17]	Netherlands	Prospective diagnostic accuracy study	CNN (version RD-174)	12-megapixel camera running either an Android 10 (Galaxy S9, Samsung, Seoul, South Korea) or iOS 13 (iPhone XR, Apple Inc., Cupertino, CA)	86.9% (82.3, 90.7)	70.4% (66.2, 74.3)	61.3% (57.9, 64.6)	90.9% (88, 93.2)	NR	76.2% (73.0, 79.1)
Birkenfeld et al. (2020) [12]	Spain	Prospective diagnostic accuracy study	Logistic regression and PCA	10-megapixel camera with camera-specific software (OLYMPUS Viewer 3, OM Digital Solutions, Tokyo, Japan)	84.0% (NR)	72.1% (NR)	58.2% (NR)	90.7% (NR)	0.89 (0.85, 0.92)	75.9% (NR)
Soenksen et al. (2021) [14]	Spain	Retrospective diagnostic accuracy study	DCNN	10-megapixel camera with camera-specific software (Olympus E-420, 14- to 42-mm lens, Olympus Corporation, Tokyo, Japan)	90.3% (90, 90.6)	89.9% (89.6, 90.2)	NR	NR	0.97 (0.969, 0.971)	86.6% (86.3, 86.8)

Quality Assessment

**Figure 2:**
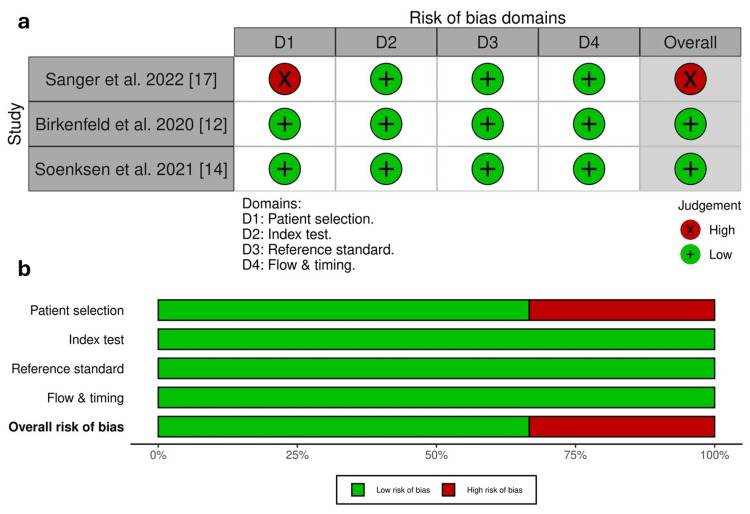
The risk of bias assessment was performed using the QUADAS-2 score to evaluate the quality of the included observational studies. Birkenfeld et al. 2020 [12], Soenksen et al. 2021 [14], and Sangers et al. 2022 [17]. D1: patient selection; D2: index test; D3: reference standard; D4: flow & timing. QUADAS-2: Quality Assessment of Diagnostic Accuracy Studies 2.

Birkenfeld et al. [12] showed high patient selection bias, while the subsequent two had a low risk of bias. However, all studies demonstrated low bias in index tests, reference standards, flow, and timing (Figure 2).

Outcomes

**Figure 3:**
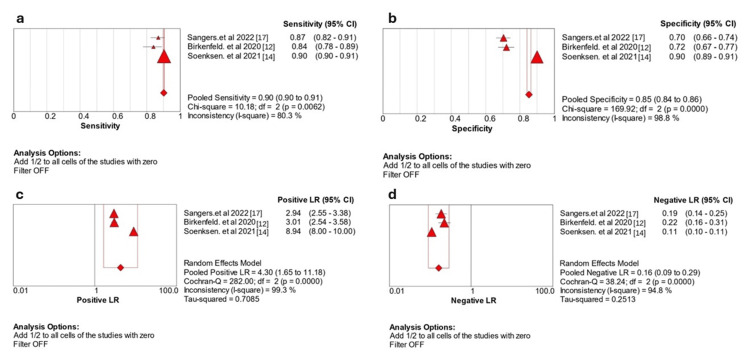
Data presents (a) a meta-analysis of sensitivity across three studies, (b) a meta-analysis of specificity across three studies, (c) a meta-analysis of positive likelihood ratios from three studies, and (d) a meta-analysis of negative likelihood ratios from three studies. Birkenfeld et al. 2020 [12], Soenksen et al. 2021 [14], and Sangers et al. 2022 [17]. LR: likelihood ratio.

The pooled sensitivity was 0.90 (95% CI: 0.90-0.91), indicating a high ability to identify true positives correctly (Figure 3A). Significant heterogeneity exists among the studies (p = 0.006, I2 = 80.3%). Likewise, the pooled specificity was 0.85 (95% CI: 0.84-0.86), indicating a strong ability to correctly identify true negatives (Figure 3B). Significant heterogeneity is observed among the studies (p = <0.001, I2 = 98.8%). In terms of PLR, the pooled effect size was 4.30 (95% CI: 1.65-11.18), indicating a moderate ability to correctly identify true positives (Figure 3C); there was substantial heterogeneity (p < 0.001, I2 = 99.3%). The pooled NLR was 0.16 (95% CI: 0.09-0.29), indicating a moderate ability to rule out the condition (Figure 3D); there was a significant heterogeneity (p < 0.001, I2 = 94.8%).

**Figure 4:**
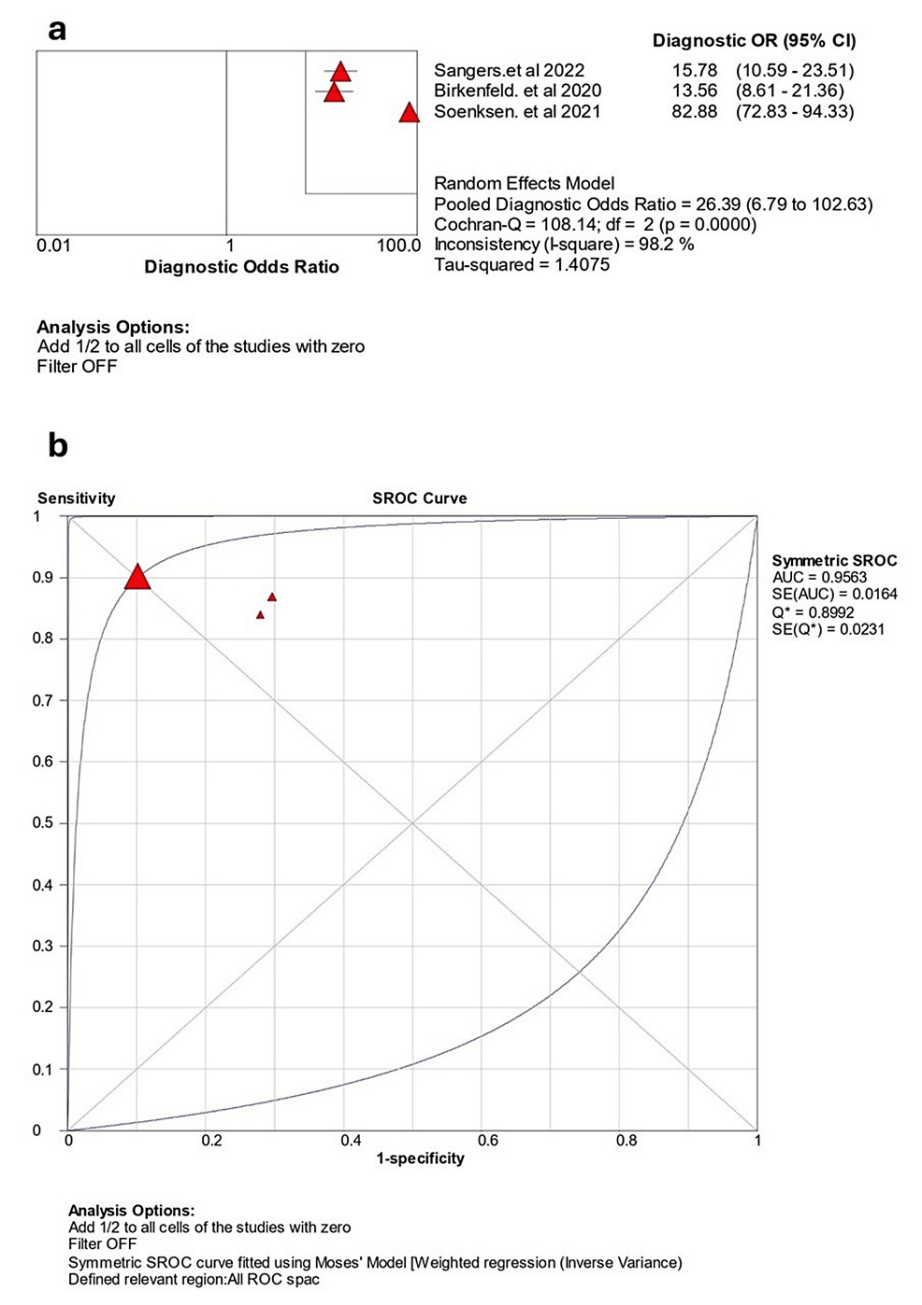
The findings of the meta-analysis of diagnostic odds ratios (a) and summary receiver operating characteristic curve (b). Birkenfeld et al. 2020 [12], Soenksen et al. 2021 [14], and Sangers et al. 2022 [17]. OR: odds ratio; SROC: summary receiver operating characteristic curve; ROC: receiver operating characteristic; AUC: area under the curve.

Figure 4A shows the findings of the DOR from three studies. The individual diagnostic ORs ranged from 15.78 to 82.88. When combined using a random effects model, the pooled DOR was 26.39 (95% CI: 6.79-102.63), indicating a substantial overall diagnostic performance. However, the studies had significant heterogeneity (p < 0.001, I2 =98.2%). The SROC curve showed that the pooled AUC was 0.9563, indicating high discriminatory power (Figure 4B).

Discussion

Our research aimed to investigate and shed light on the effectiveness of DL algorithms in the early diagnosis of patients suffering from suspicious pigmented skin lesions in primary care settings. We compared the performance of these advanced technologies with the usual diagnostic methods followed by doctors to gain a comprehensive understanding of the potential benefits of DL algorithms. The sensitivity analysis revealed the high sensitivity and specificity of the DL algorithms for detecting suspicious pigmented skin lesions in primary care settings. Our data also suggested that the DL algorithms had increased the odds of correctly diagnosing suspicious pigmented skin lesions, with an excellent discriminative ability to distinguish between benign and malignant pigmented skin lesions. Consequently, they can serve as a reliable tool for primary care physicians to detect skin cancers such as melanoma early. However, our results should be interpreted cautiously as significant heterogeneities in the pooled analyses suggest variations across the studies. This could be attributed to differences in dataset characteristics, algorithm design, lesion type variations, the doctors' expertise, or variations in the reference standard used for comparison.

Our findings align with previous studies by Birkenfeld et al. [12] and Soenksen et al. [14], who also reported significant improvements in suspicious pigmented skin lesion outcomes following similar interventions in different populations. Furthermore, Tschandl et al. also found that when an AI algorithm supports a physician's diagnosis-making, the diagnostic accuracy improves over that of either AI or physicians alone [18]. These findings have also been confirmed by other researchers [19].

Despite the promising results, there are several challenges and limitations that need to be acknowledged. Publication bias may exist, as studies with significant positive results are more likely to be published. Additionally, the quality of the included studies varied, and some may have introduced biases that could affect the overall findings. Moreover, one major limitation is the lack of interpretability of these algorithms. Hence, the accuracy of these algorithms is hard to detect when they are applied without any physician input. Besides, it is hard to explain how these algorithms come to their results. ML algorithm often functions as a black box [17] that takes in inputs and produces outputs with no interpretation of how it produced the conclusions and results. This lack of transparency can pose limitations in gaining the trust and acceptance of healthcare physicians [20]. Nevertheless, our study highlights the potential benefits of integrating AI and ML technologies, specifically DL algorithms, into routine primary care practice. These technologies can potentially enhance early detection, improve patient outcomes, and alleviate the burden on dermatologists [18]. Recently, DL methods have also been explored in the non-invasive diagnosis of skin lesions and demonstrated their ability to classify skin lesions with high accuracy. However, some other opinions believe that it is difficult to enable AI and ML technologies in daily dermatological examinations [21]. From a policy perspective, implementing large-scale skin cancer screening programs is not only likely to be a complex task but will also be infeasible in most resource-limited healthcare systems worldwide. In the United States, for example, there are fewer than 12,000 practicing dermatologists [22], and with fewer than 15 visits per 100 individuals per year [22], it is expected that most dermatology practices across the world are already too saturated and time-constrained to provide additional screening services.

Integrating DL algorithms in dermatology highlights the importance of ongoing education and training for dermatologists and primary care physicians. Continued professional development programs can help physicians stay updated with the latest technological advancements and ensure their competent use in clinical practice [20]. Collaboration between medical schools, technology companies, and hospitals can facilitate the development of specialized training programs that equip healthcare professionals with the essential skills to effectively utilize DL algorithms. A recent study by Hekler et al. found that combining humans and AI achieves a better classification of images than only dermatologists or only classification by CNN [22]. The mean accuracy increased by 1.36% when dermatologists worked together with ML and AI.

Dermatologists can contribute their expertise in curating high-quality datasets, training the algorithms, and validating their performance to optimize the use of DL algorithms in primary care settings. Primary care physicians can provide valuable insights into the practical implementation of these algorithms and ensure their integration into existing healthcare workflows. Additionally, exploring potential combination therapies involving AI and other interventions may yield synergistic effects and further enhance skin cancer outcomes.

Future research in this field should focus on refining DL algorithms to enhance their performance, reliability, and interpretability. Prospective studies with larger sample sizes and diverse patient populations are needed to validate the findings of this meta-analysis. Additionally, long-term follow-up studies can assess the impact of AI and ML algorithms on patient outcomes, including detecting early-stage skin cancers and reducing mortality rates. Human-machine collaboration has revealed promising results for future applications. For further expansion in this field, machines could assist physicians in time-consuming practices that usually are not being applied.

## Conclusions

DL algorithms have the potential to significantly improve the detection of suspicious pigmented skin lesions in primary care settings. Our analysis showed that DL exhibited promising performance in the early detection of suspicious pigmented skin lesions. However, further studies are needed. Furthermore, the integration of DL algorithms into primary care could potentially reduce the number of unnecessary biopsies and referrals, thereby optimizing resource allocation and improving patient outcomes. It is also crucial to investigate the potential challenges and limitations of DL implementation in real-world clinical settings, such as data privacy concerns and the need for standardized training datasets, to ensure its safe and effective use in dermatological practice.

## References

[1] Arnold M, Singh D, Laversanne M (2022). Global burden of cutaneous melanoma in 2020 and projections to 2040. JAMA Dermatol.

[2] Saginala K, Barsouk A, Aluru JS, Rawla P, Barsouk A (2021). Epidemiology of melanoma. Med Sci (Basel).

[3] (2024). American Cancer Society. Survival rates for melanoma skin cancer. https://www.cancer.org/cancer/types/melanoma-skin-cancer/detection-diagnosis-staging/survival-rates-for-melanoma-skin-cancer-by-stage.html.

[4] Leiter U, Keim U, Garbe C (2020). Epidemiology of skin cancer: update 2019. Adv Exp Med Biol.

[5] Gaudy-Marqueste C, Wazaefi Y, Bruneu Y (2017). Ugly duckling sign as a major factor of efficiency in melanoma detection. JAMA Dermatol.

[6] Westerhoff K, McCarthy WH, Menzies SW (2000). Increase in the sensitivity for melanoma diagnosis by primary care physicians using skin surface microscopy. Br J Dermatol.

[7] Burton RC, Howe C, Adamson L (1998). General practitioner screening for melanoma: sensitivity, specificity, and effect of training. J Med Screen.

[8] Brown AE, Najmi M, Duke T, Grabell DA, Koshelev MV, Nelson KC (2022). Skin cancer education interventions for primary care providers: a scoping review. J Gen Intern Med.

[9] Das K, Cockerell CJ, Patil A, Pietkiewicz P, Giulini M, Grabbe S, Goldust M (2021). Machine learning and its application in skin cancer. Int J Environ Res Public Health.

[10] Liu Y, Jain A, Eng C (2020). A deep learning system for differential diagnosis of skin diseases. Nat Med.

[11] Patel RH, Foltz EA, Witkowski A, Ludzik J (2023). Analysis of artificial intelligence-based approaches applied to non-invasive imaging for early detection of melanoma: a systematic review. Cancers (Basel).

[12] Birkenfeld JS, Tucker-Schwartz JM, Soenksen LR, Avilés-Izquierdo JA, Marti-Fuster B (2020). Computer-aided classification of suspicious pigmented lesions using wide-field images. Comput Methods Programs Biomed.

[13] Ding H, Zhang E, Fang F (2022). Automatic identification of benign pigmented skin lesions from clinical images using deep convolutional neural network. BMC Biotechnol.

[14] Soenksen LR, Kassis T, Conover ST (2021). Using deep learning for dermatologist-level detection of suspicious pigmented skin lesions from wide-field images. Sci Transl Med.

[15] Page MJ, McKenzie JE, Bossuyt PM (2021). The PRISMA 2020 statement: an updated guideline for reporting systematic reviews. BMJ.

[16] Whiting PF, Rutjes AW, Westwood ME (2011). QUADAS-2: a revised tool for the quality assessment of diagnostic accuracy studies. Ann Intern Med.

[17] Sangers T, Reeder S, van der Vet S (2022). Validation of a market-approved artificial intelligence mobile health app for skin cancer screening: a prospective multicenter diagnostic accuracy study. Dermatology.

[18] Tschandl P, Rinner C, Apalla Z (2020). Human-computer collaboration for skin cancer recognition. Nat Med.

[19] Han SS, Park I, Eun Chang S (2020). Augmented intelligence dermatology: deep neural networks empower medical professionals in diagnosing skin cancer and predicting treatment options for 134 skin disorders. J Invest Dermatol.

[20] Chan S, Reddy V, Myers B, Thibodeaux Q, Brownstone N, Liao W (2020). Machine learning in dermatology: current applications, opportunities, and limitations. Dermatol Ther (Heidelb).

[21] Yu L, Chen H, Dou Q, Qin J, Heng PA (2017). Automated melanoma recognition in dermoscopy images via very deep residual networks. IEEE Trans Med Imaging.

[22] Hekler A, Utikal JS, Enk AH (2019). Superior skin cancer classification by the combination of human and artificial intelligence. Eur J Cancer.

